# Cavitation fatigue in conifers: a study on eight European species

**DOI:** 10.1093/plphys/kiab170

**Published:** 2021-04-27

**Authors:** Feng Feng, Adriano Losso, Melvin Tyree, Shuoxin Zhang, Stefan Mayr

**Affiliations:** 1 College of Forestry, Northwest A&F University, Yangling, Shaanxi 712100, China; 2 Qinling National Forest Ecosystem Research Station, Huoditang, Ningshan, Shaanxi 711600, China; 3 Department of Botany, University of Innsbruck, Innsbruck 6020, Austria; 4 College of Chemistry and Life Sciences, Zhejiang Normal University, Jinhua, Zhejiang 321004, China

## Abstract

After drought-induced embolism and repair, tree xylem may be weakened against future drought events (cavitation fatigue). As there are few data on cavitation fatigue in conifers available, we quantified vulnerability curves (VCs) after embolism/repair cycles on eight European conifer species. We induced 50% and 100% loss of conductivity (LC) with a cavitron, and analyzed VCs. Embolism repair was obtained by vacuum infiltration. All species demonstrated complete embolism repair and a lack of any cavitation fatigue after 50% LC . After 100% LC, European larch (*Larix decidua*), stone pine (*Pinus cembra*), Norway spruce (*Picea abies*), and silver fir (*Abies alba*) remained unaffected, while mountain pine (*Pinus mugo*), yew (*Taxus baccata*), and common juniper (*Juniperus communis*) exhibited 0.4–0.9 MPa higher vulnerability to embolism. A small cavitation fatigue observed in Scots pine (*Pinus sylvestris*) was probably biased by incomplete embolism repair, as indicated by a correlation of vulnerability shifts and conductivity restoration. Our data demonstrate that cavitation fatigue in conifers is species-specific and depends on the intensity of preceding LC. The lack of fatigue effects after moderate LC, and relevant effects in only three species after high LC, indicate that conifers are relatively resistant against cavitation fatigue. This is remarkable considering the complex and delicate conifer pit architecture and may be important considering climate change projections.

## Teaser

The lack of fatigue effects after moderate embolism and species-specific fatigue effects after intense embolism indicate that conifers are relatively resistant against cavitation fatigue.

## Introduction

Coping with drought is fundamentally important for tree growth and survival. Water shortage in trees impairs the long-distance water transport when critical negative xylem pressure (*P*) induces cavitation and, consequently, xylem embolism and disruption of the continuity of water columns (Tyree and Sperry, 1989; Steudle, 2001; Tyree and Zimmermann, 2002). Many cases of drought-related forest mortality have already been reported (e.g. Allen et al., 2010) and such events are likely to become more frequent, as increases in duration, intensity, and frequency of dry spells, coupled with rising temperatures, are predicted to occur in the future (Meehl and Tebaldi, 2004; Schar et al., 2004; Burke et al., 2006; Jentsch et al., 2007; Kirtman et al., 2013).

Trees evolved a variety of strategies to either prevent or recover from periods of sustained drought, which are strongly related to their embolism resistance (Brodribb and Cochard, 2009; Kursar et al., 2009). These properties vary widely among species and are largely determined by differences in the structure of the xylem (Maherali et al., 2004; Sperry et al., 2006; Delzon et al., 2010). Drought-induced embolism is initiated when decreasing pressure (*P*) in the xylem causes the entry of air bubbles into functional conduits through pit membranes bordering air-filled conduits (air-seeding; Sperry and Tyree, 1988, 1990; Cochard et al., 1992; Christman et al., 2012). In recent years, the number of studies on the vulnerability of trees to drought-induced embolism and respective mechanisms of air-seeding increased (see the review by Choat et al., 2012; Choat, 2013). However, information on the xylem resistance after cycles of drought and subsequent refilling of embolized conduits is widely lacking.

For trees, which can repair their embolized conduits, embolism/repair cycles may potentially cause secondary hydraulic impairments when conduit walls or pits in the xylem are damaged due to air-seeding. As a result, the refilled conduits might be more vulnerable to embolism than when freshly produced by the vascular cambium (Hacke et al., 2001; Christensen-Dalsgaard and Tyree, 2013), and the ability of embolism repair might thus be counterbalanced by negative hydraulic legacy effects. This increased vulnerability to embolism in trees upon previous embolism events is referred to as “cavitation fatigue” (Hacke et al., 2001; Stiller and Sperry, 2002; Anderegg et al., 2013). So far, few studies observed cavitation fatigue in angiosperms, while evidence in conifers is scarce.

Among the angiosperm species which are (at least partially) capable of repairing embolism via bubble dissolution, establishing positive root/stem pressure (see review by Nardini et al., 2018) or repairing embolism under negative pressure (Bucci et al., 2003; Hacke and Sperry, 2003; Nardini et al., 2011; but also see Wheeler et al., 2013; Torres-Ruiz et al., 2015; Charrier et al., 2016), only a few species exhibited a “resilient” xylem (*Betula occidentalis*, Alder et al., 1997; *Acer negundo*, *Alnus incana*, Hacke et al., 2001; *Acer mono*, Zhang et al., 2018). Most studied species showed a reduction in embolism resistance between 1.2 and 3.0 MPa upon an embolism/refilling cycle (Sperry et al., 1991; Hacke et al., 2001; Stiller and Sperry, 2002; Melcher et al., 2003; Christensen-Dalsgaard and Tyree, 2013; Feng et al., 2015; Hillabrand et al., 2016).

The mechanism of cavitation fatigue is not well understood, but it has been hypothesized to be caused by pit membrane ruptures (when air-entry occurs due to high-pressure difference at air–water interfaces or due to rapid energy release; Hacke et al., 2001) or by stretching of membranes (due to high *P* differences before cavitation). The stretched membranes may increase the permeability of interconduit pit membranes and thus increase the probability of air-seeding (Sperry et al., 1991; Melcher et al., 2003; Christensen-Dalsgaard and Tyree, 2013; Feng et al., 2015; Hillabrand et al., 2016). Further, small bubbles left behind after embolism repair might nucleate embolism during consecutive cycles (Stiller and Sperry, 2002; but also see Hacke et al., 2001). Interestingly, the xylem sap composition might influence the extent of cavitation fatigue, both on intact plants (Stiller and Sperry, 2002) and on excised branches (Feng et al., 2015). This fatigue reduction may be related to an ionic effect on pit membrane structures (Zwieniecki et al., 2001) and/or sap surface tension (Sperry and Tyree, 1988). It might also explain observed seasonal variations in cavitation fatigue, which are linked to changes in mechanical properties of the pit membranes during development and maturation of xylem conduits and the corresponding chemical composition of the sap (Kolb and Sperry, 1999; Zhang et al., 2018). Recently, Umebayashi et al. (2019) reported that the cycling of *P* in a range even above the critical threshold for embolism formation may decrease the embolism resistance. The authors hypothesized that this “pressure fatigue” was caused by repeated mechanical stresses on the pit membranes.

Conifers, which are widely distributed, frequently studied tree species in environmental sciences and of high economic importance for forestry, exhibit a special pit architecture with pit membranes that have a torus-margo structure: on decreasing *P*, the torus is aspirated to the pit chamber aperture, operating as a sealing valve, and isolating the embolized tracheids from adjacent functional ones (Domec et al., 2006; Cochard et al., 2009; Delzon et al., 2010; Lens et al., 2013). Due to this so-called “valve effect” and relatively small and short tracheids, conifers are characterized by an overall high resistance to embolism (Hammel, 1967; Sperry and Tyree, 1990; Davis et al., 1999; Hacke and Sperry, 2001; Pittermann and Sperry, 2003; Maherali and Pockman, 2004; Bouche et al., 2014). They also operate with wider hydraulic safety margins than angiosperms (Choat et al., 2012), though they are not immune to drought-induced mortality (Breshears et al., 2005; Sanchez-Salguero et al., 2012; Hartmann et al., 2013; Savi et al., 2019). Conifer species have also been demonstrated to exhibit winter embolism (Mayr et al., 2002, 2003, 2006, 2019; Mayr and Sperry, 2010) caused by frost drought and seasonal and/or diurnal freeze/thaw cycles (Sperry and Sullivan, 1992; Mayr and Sperry, 2010). Refilling has been demonstrated to occur in some conifers after embolism by water absorption via the needle cuticle (Laur and Hacke, 2014) or bark (Katz et al., 1989; Earles et al., 2016). There is some evidence for active refilling in conifers (Borghetti et al., 1991, 1998; McCulloh et al., 2011; Klein et al., 2016; Tomasella et al., 2017), but only few data on potential cavitation fatigue. Torres-Ruiz et al. (2016) reported cavitation fatigue in *Pinus sylvestris* resulting in an embolism resistance reduction of ca. 0.5 MPa. In contrast, no cavitation fatigue was observed in alpine *Picea abies* (Mayr et al., 2020). It thus is unclear if cavitation fatigue is a relevant phenomenon in conifers, though it is an important aspect to characterize tree hydraulics and drought resistance, especially under expected climate change and the expected increase in frequency and intensity of droughts.

In this study, we tested potential cavitation fatigue in eight conifer species (Table 1). Stem segments were subjected, in a centrifuge, to *P* inducing 50% or 100% loss of conductivity (LC), before embolism was repaired by vacuum infiltration. A potential bias due to aspirated pits and the resulting reduction in absolute hydraulic conductivity was considered. Cavitation fatigue was quantiﬁed by how much the vulnerability curve (VC) measured after embolism/repair was shifted compared to before embolism. Potentials shifts were quantified based on the shift in *P*_50_ (Δ*P*_50_), which is *P* inducing 50% LC. VC defines the relationship between decreasing *P* and corresponding LC for a given species, and *P*_50_ is the most important threshold used to compare embolism resistance among species (Choat et al., 2012). We hypothesized species-specific shifts in VCs (i.e. cavitation fatigue), especially after repair of high conductivity losses.

**Table 1:** List of study species and respective growth type, sampling location, and elevation

Species	Growth Type	Site of Harvest	Elevation (m)
*L. decidua*	Tree, deciduous	Praxmar, 47°09′ N, 11°07′ E	2,100
*P. cembra*	Tree, evergreen	Praxmar, 47°09′ N, 11°07′ E	2,100
*P. mugo*	Shrub, evergreen	Birgitz Köpfl, 47°11′ N, 11°19′ E	2,035
*P. sylvestris*	Tree, evergreen	Innsbruck, 47°16′ N, 11°22′ E	600
*P. abies*	Tree, evergreen	Innsbruck, 47°16′ N, 11°22′ E	600
*A. alba*	Tree, evergreen	Innsbruck, 47°16′ N, 11°22′ E	600
		Gallzein, 47°21′ N, 11°46′ E	950
*T. baccata*	Tree, evergreen	Innsbruck, 47°16′ N, 11°22′ E	600
		Gallzein, 47°21′ N, 11°46′ E	950
*J. communis*	Shrub, evergreen	Zirl, 47°16′ N, 11°16′ E	740

## Results

### Embolism repair

All samples, regardless of species, exposed to a *P* inducing 50% LC, showed complete repair of conductivity after vacuum infiltration for 12 h (99.56% ± 1.84% of initial specific xylem hydraulic conductivity, *K*_s_, was restored; Table 2). Samples exposed to a *P* inducing 100% LC reached 97.80% ± 1.51% of initial *K*_s_ after 24 h of vacuum infiltration, in all species except *P. sylvestris* (Table 2). In the latter, only ca. 65% of initial *K*_s_ could be restored.

**Table 2:** Restoration of *K*_s_ (i.e. percentage recovery of the conductivity after one cycle of embolism and repair) and *P*_50_ before (*P*_50, before_) and after (*P*_50, after_) inducing 50% and 100% LC

Species	50% LC Treatment			100% LC Treatment		
	Restoration of *K*_s_ (%)	*P*_50, before_ (MPa)	*P*_50, after_ (MPa)	Restoration of *K*_s_ (%)	*P*_50, before_ (MPa)	*P*_50, after_ (MPa)
*L. decidua*	108.95 ± 6.33 (7)	−3.66 ± 0.08	−3.75 ± 0.08	97.88 ± 6.09 (6)	−3.75 ± 0.09	−3.78 ± 0.10
*P. cembra*	108.95 ± 5.40 (6)	−3.57 ± 0.02	−3.49 ± 0.02	96.79 ± 3.11 (6)	−3.58 ± 0.07	−3.53 ± 0.09
*P. mugo*	95.92 ± 4.29 (4)	−4.49 ± 0.07	−4.47 ± 0.06	93.14 ± 4.50 (6)	−4.53 ± 0.11	−4.14 ± 0.08^*^
*P. sylvestris*	98.20 ± 8.30 (7)	−3.58 ± 0.05	−3.59 ± 0.08	65.87 ± 7.04 (12)	−3.59 ± 0.07	−3.22 ± 0.09^*^
*P. abies*	101.60 ± 5.19 (8)	−3.63 ± 0.04	−3.68 ± 0.06	95.38 ± 5.07 (8)	−3.67 ± 0.05	−3.57 ± 0.07
*A. alba*	94.26 ± 4.28 (6)	−3.69 ± 0.04	−3.72 ± 0.05	104.99 ± 3.29 (4)	−3.53 ± 0.18	−3.50 ± 0.14
*T. baccata*	93.97 ± 0.68 (6)	−6.62 ± 0.10	−6.84 ± 0.21	95.91 ± 1.52 (5)	−6.70 ± 0.16	−5.78 ± 0.12^*^
*J. communis*	95.92 ± 3.91 (6)	−5.92 ± 0.17	−6.29 ± 0.24	99.11 ± 3.50 (4)	−6.06 ± 0.13	−5.39 ± 0.28^*^

Data are given as mean ± se, sample replicates in each treatment are shown in parentheses.

* Indicates signiﬁcant differences between *P*_50_ obtained from VCs measured before and after embolism-repair cycles at a probability level of 5% (Student’s *t* test).

### Vulnerability analyses

Among the harvested species, the highest resistance to embolism was observed in *Taxus baccata* and *Juniperus communis*, with *P*_50_ of −6.70 ± 0.16 MPa and −6.06 ± 0.13 MPa, respectively, followed by *Pinus mugo*, with *P*_50_ of −4.53 ± 0.11 MPa, while the most vulnerable species were *Pinus cembra*, with *P*_50_ of −3.58 ± 0.07 MPa; *P. sylvestris*, with *P*_50_ of −3.59 ± 0.07 MPa; and *Abies alba*, with *P*_50_ of −3.53 ± 0.18 MPa (Table 2).

**Figure 1:**
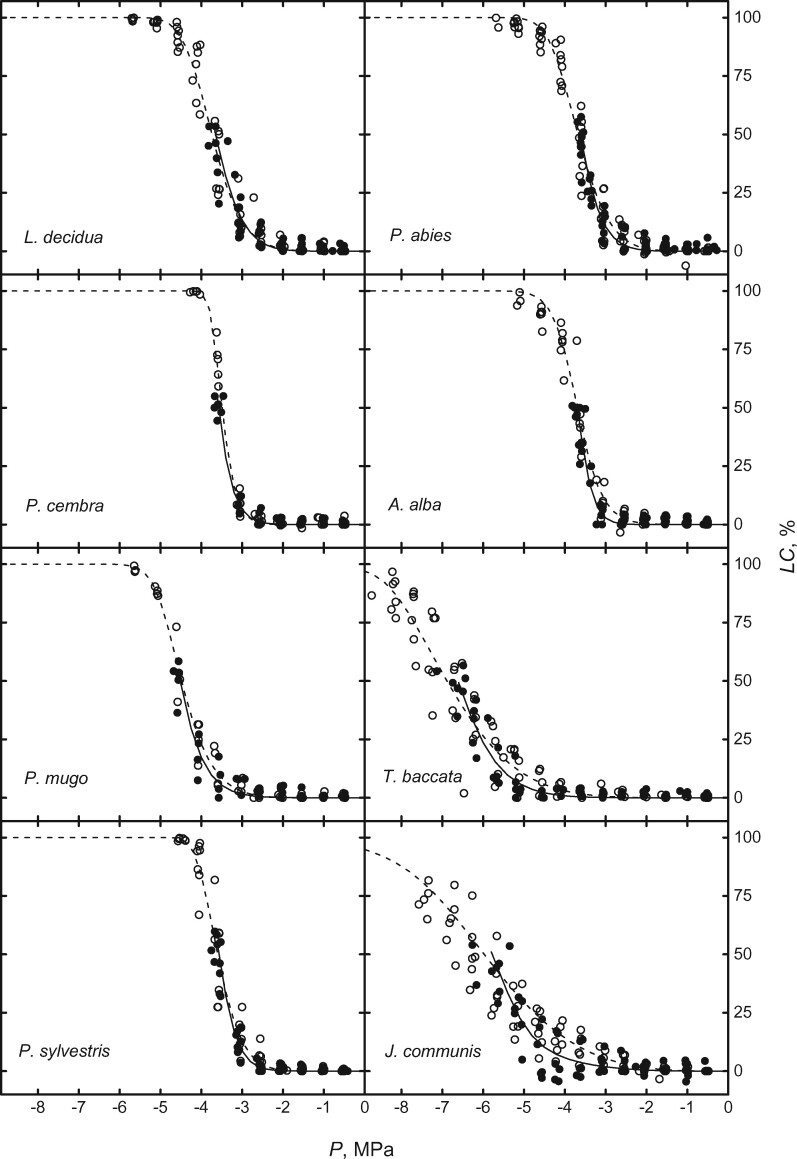
Plotted mean VCs measured on stem segments before (filled symbols and solid curves) and after (with previous embolism repair, open symbols, and dashed lines) induction of 50% LC. Note that the first VCs end at 50% LC, when embolism was removed before the second VCs were measured.

**Figure 2:**
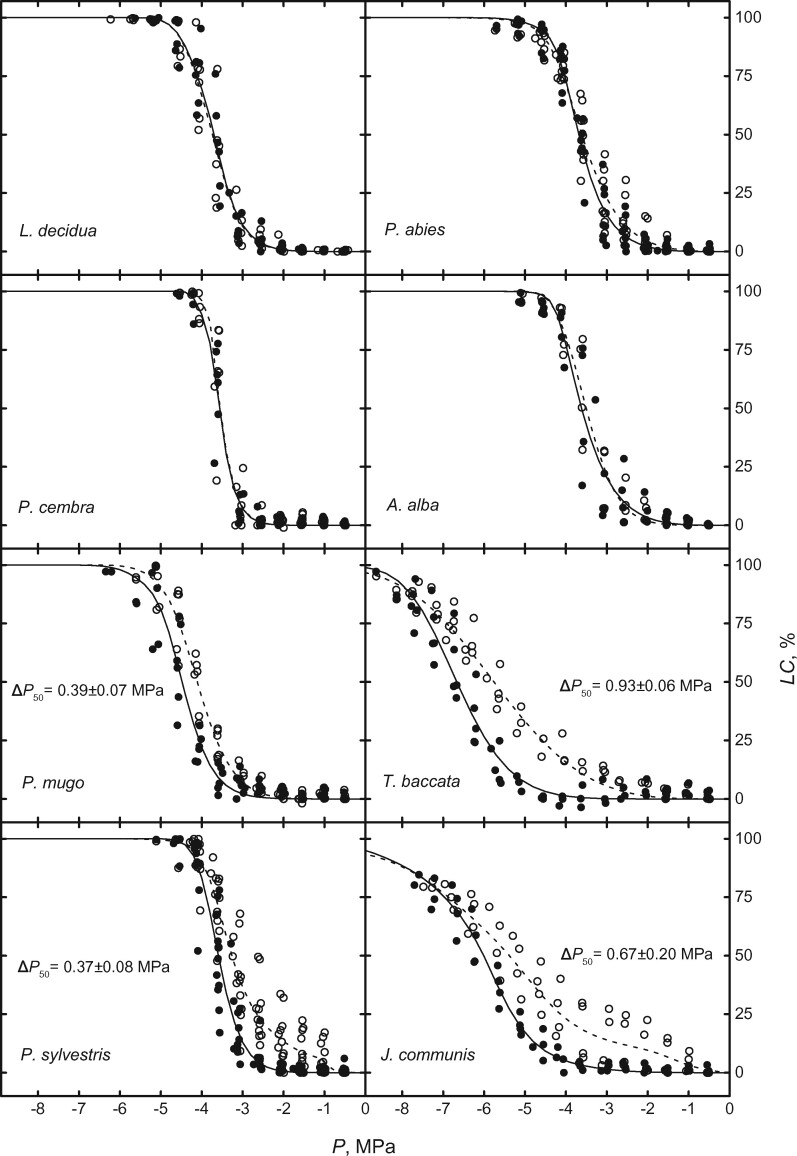
Plotted mean VCs measured on stem segments before (filled symbols and solid curves) and after (with previous embolism repair, open symbols, and dashed lines) induction of 100% LC. The first VCs end at 100% LC, when embolism was removed before the second VCs were measured. Mean Δ*P*_50_ ± se is given only if the difference between *P*_50_ of the two curves is significantly different (*P* < 0.05, Student’s *t* test).

**Figure 3:**
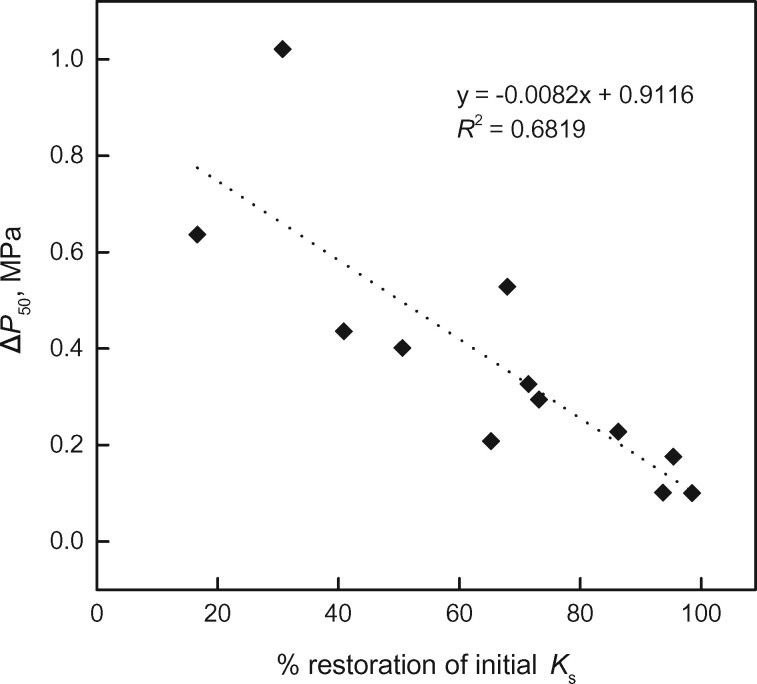
Correlation of cavitation fatigue (Δ*P*_50_, i.e. shift in *P* inducing 50% LC after induction of 100% LC) versus percentage of restoration of initial *K*_s_ by vacuum infiltration in *P. sylvestris*. The dotted line indicates significant correlation based on a linear regression of plotted values (*P* = 0.001).

After induction of 50% LC and repair of induced embolism by vacuum infiltration, no difference in VCs before and after treatment was observed in any of the study species (Figure 1; Table 2; 50% LC treatment). This indicated a lack of cavitation fatigue upon medium (i.e. 50% LC) embolism. After induction of 100% LC, a cavitation fatigue was also absent in *Larix decidua*, *P. cembra*, *P. abies* and *A. alba* (Table 2; Figure 2; 100% LC treatment). In contrast, the other species showed increased vulnerability to embolism with the less negative *P*_50_. Δ*P*_50_ in *P. mugo*, *P. sylvestris*, *T. baccata*, and *J. communis* were 0.39 ± 0.07 MPa, 0.37 ± 0.08 MPa, 0.93 ± 0.06 MPa, and 0.67 ± 0.20 MPa, respectively. In the case of *P. sylvestris*, the shift of *P*_50_ before and after 100% LC induction was negatively correlated with the fraction of initial *K*_s_ restored by vacuum infiltration (Figure 3).

## Discussion

A combination of vulnerability analyses, performed with the cavitron technique, and embolism repair, induced by vacuum infiltration, enabled an efficient testing of potential cavitation fatigue in conifer species under study. After 50% LC no cavitation fatigue was observed, while induction and repair of 100% LC led to a shift in the VC in four out of eight study species. A prerequisite for successful analyses was complete embolism repair between VC measurements, which was obtained in all species, except *P. sylvestris*. In the following, embolism repair and observed species-specific cavitation fatigue are discussed.

### Embolism repair

The complete restoration of initial *K*_s_, as found in nearly all study species (Table 2), demonstrates that pit aspiration, induced by low *P*, was reversible (Edwards et al., 1994; Mayr et al., 2020). Otherwise, decreased *K*_s_ would have been observed due to the sealing of the torus against the pit aperture (Sperry and Tyree, 1990). The ability of pit membranes to return to their relaxed positions corresponds to field observations on conifers at the alpine treeline, where the number of aspirated pits and LC corresponded during winter, when embolism was formed and repaired, was demonstrated (Mayr et al., 2014, 2019). It is surprising that *P* leading to 100% LC even at ca. −7 to −8 MPa (in *T. baccata* and *J. communis*; Figure 2), and thus to failure of the sealing mechanism, did not affect *K*_s_ restoration. This result indicates that vacuum infiltration is an effective method to restore conductivities in conifers, and it is probably more efficient than flushing (Sperry and Tyree, 1990). The complete restoration of initial *K*_s_ also demonstrates that this technique avoids resin clogging (a frequent problem with flushing following Sperry et al., 1988). And it indicates that even very negative *P* did not cause membranes to be severely stretched beyond their elastic limit. In this case, an increase in *K*_s_ would have been observed after vacuum infiltration (Cochard et al., 2009), as it would be caused by either the breakage of the membrane (rupture; Sperry and Tyree, 1990) or permanent slippage of the torus from its sealed position (permanent deformation; Domec et al., 2006). Accordingly, it is also unlikely that small microbubbles were left behind (Hacke et al., 2001; Stiller and Sperry, 2002).

### Vulnerability measurements

In our study, low *P* was induced artificially in a centrifuge by using the cavitron method (i.e. flow centrifuge technique). Like all other hydraulic methods, the cavitron method cannot completely mimic the situation in natura, but this method is a time- and material-efficient technique, and it allows flow measurements under negative potential (Cochard et al., 2005). Furthermore, this method is well-established for analyses on conifers and short-vessel species, and many studies showed good agreements between the centrifuge technique and other methodical approaches (Sperry method, micro X-ray, and acoustic emissions). A disadvantage of the centrifuge technique is that it creates a parabolic water potential gradient across the sample (with the target potential reached only in the center), which may slightly bias the resulting VCs. Fortunately, the latter is not of relevance in our study, which compares vulnerability to embolism before and after refilling: in both cases, conductivity measurements reflect embolism formation in the segment’s center, and thus allow reliable quantification of potential cavitation fatigue.

It has to be noted that branch samples used in the cavitron contained several tree rings, which may have influenced measured cavitation fatigue: older tree rings were probably exposed to embolism and repair during previous winters, and thus to fatigue, which led to a shift of the first measured VC. However, the current tree ring, which contributes most to hydraulic conductance, was not prestressed and, accordingly, not affected by previous fatigue. We thus expect the overall effect of previous fatigue to be small and previously stressed samples to exhibit only slightly higher fatigue.

Almost identical VCs were obtained on samples before and after exposure to *P* at 50% LC (Figure 1), indicating absence of cavitation fatigue in study species, though embolism resistance differed considerably across species (Table 2; P_50, before_). This indicates that pit structures responsible for hydraulic safety were not affected at moderate *P* and LC. A lack of cavitation fatigue was also found after 100% LC in *L. decidua*, *P. cembra*, *P. abies*, and *A. alba* (Figure 2), while *P. mugo*, *P. sylvestris*, *T. baccata*, and *J. communis* showed less resistance to embolism. With respect to literature, findings on *P. abies* confirm the lack of cavitation fatigue (Mayr et al., 2020) for trees from the alpine treeline, which undergoes annual winter embolism (up to 100% LC) and repair (Mayr et al., 2002, 2006). The observed small cavitation fatigue of *P. sylvestris* with a Δ*P*_50_ of ca. 0.4 MPa (Figure 2) is in accordance with the fatigue of ca. 0.5 MPa reported by Torres-Ruiz et al. (2016). However, this result might be biased by incomplete embolism repair: variable rates of restoration in *K*_s_ after repair process were observed from 16.65% to 98.49% (Figure 3), which influenced *P*_50_ in the second vulnerability measurements, resulting in “artificial” cavitation fatigue. Remaining air in the xylem might have caused nucleation of embolism at less *P* (Hacke et al., 2001; Stiller and Sperry, 2002). It is thus likely that *P. sylvestris* does not exhibit a pronounced cavitation fatigue if embolism repair is successful. Overall, we found significant cavitation fatigue (after high LC, *P* < 0.05; Table 2) in at least three out of eight species. In the following, we discuss (1) the relevance of observed cavitation fatigue in natura and (2) its possible structural causes.

(1) It is known that some tree species growing at the alpine treeline undergo annual winter embolism/repair cycles. Mayr et al. (2006) reported high LC and low water potentials during winter in *P. mugo* (>80% LC, approximately −2 MPa) and *J. communis* (>80% LC, approximately −6.5 MPa), which both showed cavitation fatigue in our study (Figure 2). In *P. mugo*, the only moderate negative water potential was probably due to rehydration of twigs below the snow, which can lead to an increase in water potentials within a few days, while embolism repair requires several weeks (Mayr et al., 2019). It is thus likely that these two species, at least at treeline sites, can suffer from weakened xylem due to previous embolism. A combination of freezing/thawing and drought (“frost drought,” Mayr et al., 2006) may amplify potential cavitation fatigue as Feng et al. (2015) demonstrated similar fatigue effects after freezing/thawing and drought/rehydration cycles. The genus *Juniperus* is known to exhibit impressively negative vulnerability thresholds, nevertheless it can be prone to native embolism (e.g. Mayr et al., 2006; West et al., 2008; Johnson et al., 2018). Thus, even conifer species with low vulnerability may be affected by cavitation fatigue, and shifts of nearly 1 MPa in *P*_50_, as observed in *J. communis* and *T. baccata* (Figure 2), may be lethal under repeated severe droughts. The other conifers under study did not exhibit critical water potentials in previous field studies. In Mayr et al. (2006), for instance, the lowest water potential was above −2.5 MPa and the highest LC below 20% in *P. cembra* and *L. decidua*. However, due to climate change, prolonged and more severe drought periods are expected in the future, which may cause lower water potential and higher risk of embolism, especially at higher elevations (Barry, 2008; Marty and Meister, 2012; Ohmura, 2012; Jiménez Cisneros et al., 2014; Kovats et al., 2014; Wang et al., 2016). In consequence, the relevance of cavitation fatigue will increase, although effects after repeated droughts and respective embolism/repair cycles are unclear: Feng et al. (2015) found that a reduced embolism resistance was only induced in the first out of four cycles of embolism and refilling. In contrast, Umebayashi et al. (2019) showed that repeated ﬂuctuation of sap *P*, even above the critical thresholds for embolism formation, can weaken the xylem. It is important to note that these hydraulic impairments may cause long-term effects, as demonstrated in Anderegg et al. (2013), who reported hydraulic impairments in aspen 8 years after the initial drought, and in Sperry et al. (1991), who found a degradation of embolism resistance in ageing xylem of *Populus*.

It should also be mentioned that laboratory experiments on cavitation fatigue did not perfectly simulate field situations, as embolism formation and repair normally occur over much longer time spans in the latter. This may cause a more stable aspiration of pits and more difficult reopening. Field studies would be required to estimate the relevance of experimentally demonstrated cavitation fatigue in natura.

(2) From an anatomical point of view, it is likely that observed cavitation fatigue was related to pit structures, which play a role in potential air-seeding (Hacke et al., 2001; Stiller and Sperry, 2002). The stability of the torus aspiration depends on a ratio between torus and pit aperture diameter (i.e. torus overlap; Domec et al., 2006; Cochard et al., 2009; Delzon et al., 2010; Bouche et al., 2014). Accordingly, *P. mugo* and *T. baccata*, which have a relatively small torus overlap (Delzon et al., 2010), showed cavitation fatigue (Figure 2). However, *J. communis*, which also showed pronounced cavitation fatigue, has a rather broad torus overlap (Delzon et al., 2010) and thus other pit structures, such as damages on the pit aperture or pit chamber (Cochard et al., 2009; Delzon et al., 2010) and/or increased porosity of the torus (Jansen et al., 2012), might be involved in an increased risk of air-seeding. Furthermore, loosening of margo strands may lead to larger pores (Sperry et al., 1991; Hillabrand et al., 2016) and/or allow the torus to slip off the aspiration position (Sperry and Tyree, 1990; Domec et al., 2006) and, in consequence, cause air-seeding at less negative *P*. Tracheid cell walls in conifers contain varying amounts of cellulose, hemicellulose, and lignin, and variation in this composition might influence, for example, the membrane flexibility and thus their species-specific variation in embolism resistance and potential cavitation fatigue (Domec et al., 2006). Compositions of pit membranes may also show seasonal changes during development and maturation of xylem conduits (Kolb and Sperry, 1999), and the chemical composition of the xylem sap may change as well (Losso et al., 2017, 2018; Schenk et al., 2017). Further studies are required to understand the underlying, and perhaps multifold, structural changes leading to cavitation fatigue in conifers, though identifying pits responsible for air-seeding will be challenging.

## Conclusion

Some conifers under study showed impressive resistance against cavitation fatigue, while some species showed shifts in vulnerability of up to 0.9 MPa after an induction of 100% LC. This is substantial, though still lower than cavitation fatigue found in most angiosperms. Observed species-specific responses to embolism/repair cycles may be relevant with respect to climate change and the expected increased frequency in drought events. From an anatomical point of view, it is fascinating that the complex and delicate pit architecture of most conifers can withstand embolization and repair without substantial damage.

## Materials and methods

### Plant material

Experiments were performed on eight forest conifer species growing in Tyrol, Austria, Central European Alps (Table 1). The sampling was conducted from October to March in 2018/2019, because winter is the crucial season for Alpine conifers when large amounts of embolism can occur (Mayr et al., 2002, 2003, 2006, 2019, 2020) and cavitation fatigue might play a role in natura. Samples were collected right before the formation of winter embolism (expecting no or minor embolism). For measurements, branches (identical age within species) of similar size with lengths of 60–120 cm were harvested, wrapped in black plastic bags, and transferred to the laboratory. After releasing the internal tension by re-cutting the basal ends (by ca. 10 cm in total) under tap water, branches were allowed to rehydrate overnight in buckets filled with tap water and wrapped in black plastic bags.

A 28-cm-long stem segment with basal diameter of approximately 6 mm was cut from the main stem of each branch while submerged in tap water, starting at approximately 20 cm from the apex. The stem end, which was submersed during rehydration, was not included. Leaves, side branches, and bark of the entire sample were removed. As the majority of resin ducts is located in the bark, its removal is important to reduce potential resin clogging and respective bias in vulnerability measurements. The segment was then trimmed under tap water to 27.4 cm in length with a sharp carving knife, which was sharpened multiple times between cuttings. The segment was then exposed to embolism (induced and analyzed via cavitron) and repair cycles.

### Cavitron measurements

Vulnerability measurements were performed with the cavitron technique (Cochard et al., 2005; Beikircher et al., 2010). Stem segments were exposed in the centrifuge to stepwise decreasing *P* until 50% and 100% LC were reached. From *P* and corresponding LC data, the first VCs were constructed (whereby VCs from 50% LC samples showed only half of the potential entire curve). All stem segments were subsequently subjected to vacuum inﬁltration to remove previously induced embolism. After cutting off ca. 1-mm-thick slices at both ends to remove potential resin layers, the segments were again exposed to decreasing *P* until 100% LC was reached for construction of the second VC.

Briefly, 27.4-cm-long stem segments were fixed in a custom-built rotor (Cochard, 2002) mounted on the Sorvall RC-5 centrifuge (DuPont Instruments, USA). Both ends of the segments were placed in reservoirs filled with distilled, filtered (0.22 μm) and degassed water containing 0.005% (v/v) “Micropur Forte MF 1000F” (Katadyn Products, Wallisellen, Switzerland). The temperature in the chamber of the centrifuge was set to 10°C. Samples were equilibrated for at least 20 min at −0.25 MPa before measurements. A pressure gradient (Δ*P*) generated by different amounts of water in the two reservoirs (upstream and downstream reservoir, respectively) drove the water flow through the segment. Both of the menisci in the two reservoirs could be observed using a camera (Motic 1SP, Motic Deutschland GmbH, Wetzlar, Germany). Then, the rate of the water movement (*F*) in the segment was directly measured by calculating the time interval for a certain displacement of the moving meniscus in the upstream reservoir toward the downstream reservoir. The *K*_s_ at the current Δ*P* was calculated in Eq. (1) as follows:

(1) $$K_{s}=\frac{F\;L}{\Delta P\;A}$$

where *L* was the length and *A* was the xylem area of the stem segment spinning in the cavitron.

Followed by 20-min equilibration at −0.25 MPa, the maximum conductivity (*K*_max_) of the stem segment was measured after 2-min stabilization at −0.5 MPa. Subsequently, a VC was obtained by repeated conductivity measurements with a stepwise increase in RPM at intervals of 0.5 MPa, subjecting the stem segment to decreasing *P* until LC reached 50% or 100%. LC was calculated in Eq. (2) as follows:

(2) $$LC=100\times \left(1-\frac{K_{s}}{K_{\max}}\right)\;$$

VCs were sigmoidal and fitted to a Weibull function (cumulative distribution function; Cai et al., 2010, 2014; Wang et al., 2014) in Eq. (3) as follows:

(3) $$\mathrm{LC}/100=1+\text{exp}[-{\left(P/B\right)}^{C}]$$

where *B* and *C* were the Weibull constants that were calculated by minimizing *RMS*_error_.

In the whole measurement process, flow rates over the sample were low. It is thus unlikely that resin, eventually released from opened ducts in the xylem, was pushed into conduits (as it might happen during high-pressure flushing of the “Sperry method”; Sperry et al., 1988).

### Embolism repair

The vacuum infiltration method (Pivovaroff et al., 2016; Mayr et al., 2020) was used to rehydrate stem samples after exposure to 50% and 100% LC. This method would neither force the resin out of ducts nor into conduits, unlike high-pressure flushing of the “Sperry method” (Sperry et al., 1988). Embolized samples were immersed in the solution used to measure *K*_s_ and VCs (please see above “cavitron measurements”) and placed under a partial vacuum of −850 mbar generated by a pump (N035 AN. 18, KNF Neuberger GmbH, Freiburg, Germany). Fifty percent of LC segments were vacuum infiltrated for 12 h, while 100% of LC samples were infiltrated for 24 h and then stored under water overnight in the fridge (4°C). According to preliminary tests, the time of vacuum infiltration and submersion were necessary and sufficient to repair respective LC.

### Statistics

Values are given as mean ± se. Student’s *t* test was used to test for differences between two groups. All statistical analyses were done with SPSS software version 18.0 (SPSS Inc., Chicago, IL, USA) at a probability level of 5%.

## Notes

S.M., M.T., and F.F. designed the research project. F.F. and A.L. performed data collection, F.F. did data analyses. F.F., A.L., S.M., M.T., and S.Z. interpreted the data and prepared the manuscript.

The author responsible for distribution of materials integral to the findings presented in this article in accordance with the policy described in the Instructions for Authors (https://academic.oup.com/plphys/pages/general-instructions) is: Shuoxin Zhang (sxzhang@nwafu.edu.cn).
